# Neuroleptic Malignant Syndrome: A Case Aimed at Raising Clinical Awareness

**DOI:** 10.1155/2015/769576

**Published:** 2015-06-11

**Authors:** Jad Al Danaf, John Madara, Caitlin Dietsche

**Affiliations:** Department of Internal Medicine, Thomas Jefferson University Hospital, 1025 Walnut Street, Jefferson College Building, Suite 805, Philadelphia, PA 19107, USA

## Abstract

A 60-year-old man with a history of bipolar disorder on risperidone, bupropion, and escitalopram was admitted for community acquired streptococcal pneumonia. Four days later, he developed persistent hyperthermia, dysautonomia, rigidity, hyporeflexia, and marked elevation of serum creatine phosphokinase. He was diagnosed with neuroleptic malignant syndrome (NMS) and improved with dantrolene, bromocriptine, and supportive therapy. This case emphasizes the importance of considering a broad differential diagnosis for fever in the ICU, carefully reviewing the medication list for all patients, and considering NMS in patients with fever and rigidity.

## 1. Case Presentation

A 60-year-old man with a past medical history of hypertension, type II diabetes mellitus, dyslipidemia, coronary artery disease, obstructive sleep apnea, depression, and bipolar disorder controlled on bupropion, escitalopram, risperidone, and valproate initially presented to his primary care provider's office with a chief complaint of shortness of breath, cough, and confusion. The patient was sent to the emergency department (ED) for further evaluation.

In the ED, the patient initially appeared in mild respiratory discomfort. The temperature was 37.1°C (98.8°F), the pulse 106 beats per minute, the blood pressure 83/60 mmHg, the respiratory rate 20 breaths per minute, and oxygen saturation 87% requiring high flow nasal cannula. He continued to desaturate and was placed on Bi-PAP before requiring intubation for hypoxic respiratory failure. The physical examination was only notable for crepitations to auscultation over the right lateral anterior chest examination. The initial chest X-ray showed a large consolidation at the right lung base. Blood, urine, and sputum cultures were sent and the patient was started on broad spectrum antibiotics including vancomycin, piperacillin-tazobactam, and azithromycin for severe sepsis secondary to community acquired pneumonia. He was then transferred to the medical intensive care unit (MICU) for further management.

## 2. Hospital Course and Diagnosis

In the MICU, the patient was sedated with a continuous fentanyl infusion and lorazepam as needed for agitation. Blood cultures, urine culture, sputum cultures, urine* Legionella*, and streptococcal antigen tests were pending. The patient was febrile at 39.3°C on day 1 of hospitalization. His home medications including bupropion, valproate, escitalopram, and risperidone were continued.

On day 2, both sets of blood cultures obtained on admission grew* Streptococcus pneumoniae* and the urine streptococcus antigen was positive. On day 3 of admission, the sputum culture showed very few Gram positive cocci and the subsequent blood cultures cleared. Once susceptibilities were known, the antibiotics were narrowed to ceftriaxone on day 2.

On day 4 of admission, the patient was persistently febrile up to 40.5°C, hypertensive at 160/90 mmHg, and tachycardic at 110 beats per minute. On physical examination, the patient was intubated and sedated, appeared more agitated, and had flushed skin and was diaphoretic. The oral mucous membranes were dry and the neck was supple. No peripheral edema was noted and he had normal capillary refill. There were persistent crepitations at the right base on anterior chest auscultation and the heart exam was normal with normal S1 and S2 and regular rate and rhythm at 110 beats per minute with no murmurs, rubs, or gallops. On neurologic examination, his pupils were equal in size and minimally reactive to light. He was rigid symmetrically in all extremities (shoulders, elbows, wrists, hips, knees, and ankle joints) and hyporeflexic with no clonus. Kernig and Brudzinski's signs were negative with no neck rigidity. The repeat chest X-ray showed mild resolution of the right lower lobe consolidation. The blood cultures had cleared, and repeat cultures did not grow any organism in the blood or urine. Due to rigidity, a blood creatine phosphokinase (CPK) level was checked and it was 8,450 mcg/L (10–120 micrograms per liter), and despite aggressive fluid resuscitation, it continued to trend upwards reaching a peak of more than 12,000 mcg/L (10–120 mcg/L) on the same day. A central nervous system infection was unlikely given that the patient did not have neck rigidity or mental status changes at that point.

Given that most common infectious causes for these symptoms had been ruled out, we considered other causes of fever including neuroleptic malignant syndrome (NMS), serotonin syndrome (SS), malignant hyperthermia, and nonconvulsive seizures. A 48-hour continuous EEG monitoring showed diffuse background slowing with no epileptiform activity; hence NMS or SS was the most likely diagnosis.

The following medications were stopped starting day 5 of admission due to their correlation with NMS (risperidone) and SS (escitalopram, valproate, bupropion, and fentanyl). Other associated lab abnormalities were also noticed including leukocytosis at 17,400 wbc/mcl (4,500–10,000 white blood cells per microliter), hypernatremia at 149 mEq/L (135–145 milliequivalents per liter), mild hypocalcemia with a corrected serum calcium at 7.8 mg/dL (8.5–10.2 milligrams per deciliter), moderately elevated liver transaminases, and lipase. Dantrolene (120 mg intravenous one dose changed to 100 mg per nasogastric tube every 8 hours) and bromocriptine (5 mg per nasogastric tube every 8 hours increased to 10 mg per nasogastric tube every 8 hours) were started for NMS. For fever reduction, we used a combination of intravenous fluids, ibuprofen, acetaminophen, cooling blankets, ice packs, free water flushes into the stomach via oral-gastric tube, and low dose lorazepam as needed.

**Figure 1:**
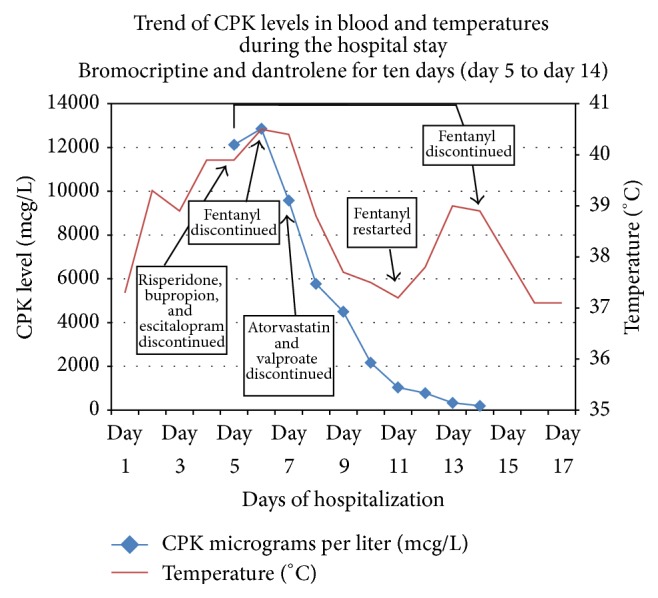
The trend in the serum level of creatine phosphokinase (CPK) in micrograms/liter (mcg/L) and body temperature in degrees Celsius (°C) across the hospital stay, with highlights on when certain medications were discontinued in correlation with these trends.

On day 9, the patient was less agitated, cooperative, and following commands but was still dependent on the ventilator. His fever curve was improving, he was noted to be less rigid, and his CPK levels were trending downwards. Dantrolene and bromocriptine were continued for a total of 10 days. It is noteworthy that fentanyl was restarted on day 11 for mild sedation, after which the patient had a fever, which subsided after stopping the medication the next day.  Figure 1 presents the trend of CPK levels and temperature variations throughout the hospital stay, highlighting the days when most of the above mentioned medications were discontinued.

On day 17, the patient was discharged to a long term acute care facility for tracheostomy care for a week and subsequently home after decannulation, returning to his baseline level of functioning and back to work.

## 3. Discussion

NMS is an uncommon, yet life threatening, condition that is frequently missed in patients who present with fevers [1]. As in our patient, new onset fevers without a definitive etiology led us to expand our differential diagnosis to include noninfectious causes. While carefully reviewing his medication list, we noticed that he was on multiple chronic and acute medications that have been identified as triggering agents in NMS and SS. High potency, first generation antipsychotics such as haloperidol are most commonly indicated in causing NMS though other agents have also been indicated including atypical antipsychotics and centrally acting antiemetic agent [2, 3]. SS is precipitated by a more extensive list of drugs including selective serotonergic reuptake inhibitors, anticonvulsants, antiemetics (ondansetron and metoclopramide), antibiotics (linezolid), cold remedies (dextromethorphan), and drugs of abuse (ecstasy and LSD). Our patient was getting bupropion, valproate, escitalopram, and fentanyl which can all be causative agents in SS. Given how clinically similar SS is to NMS, we held these medications as well.

The pathogenesis behind the cause of NMS is only speculative and has not yet been clearly defined [1]. NMS has a unique picture that is usually characterized by four clinical findings: fever, rigidity, altered mental status, and autonomic instability [4, 5]. The classical “lead pipe” rigidity is noticed on physical exam [5] which is defined by increased resistance to all ranges of motion in all extremities. Hyperthermia greater than 38°C is seen in the majority of cases but sometimes can reach greater than 40°C. Autonomic dysregulation, including tachycardia, hypertension, and tachypnea, is seen. This pattern of clinical findings is not entirely specific and can be seen with multiple medical conditions leading to a broad differential diagnosis. In particular, serotonin syndrome (SS) presents with similar symptoms to NMS including hyperthermia, autonomic instability, and mental status changes [6]. In this setting, laboratory abnormalities can be identified to increase the clinical suspicion of NMS [7, 8]. Elevated serum creatine kinase levels are most commonly seen and are typically greater than 1,000 mcg/L but can reach up to 10,000 mcg/L (10–120 mcg/L) or higher in severe NMS [9, 10]. A multitude of other laboratory abnormalities including leukocytosis and electrolyte disturbances can be caused by NMS. Furthermore, NMS develops over days to weeks, whereas SS develops over hours to days. NMS is characterized by a sluggish neuromuscular response such as rigidity and hyporeflexia; and SS is characterized by neuromuscular hyperactivity involving tremor, myoclonus, and hyperreflexia [2].

Once NMS is suspected, several management options are available. First it is important to stop the suspected causative agents and keep the patient well hydrated to prevent acute renal failure. Autonomic instability can be managed with antihypertensive agents and anxiolytics. The use of antipyretics is not much supported by evidence in the literature but it has been used in several situations to treat hyperthermia. Medications can be used to directly treat NMS though none of them have been tested in large clinical trials and are only used based on recommendations from expert opinions. Dantrolene, a skeletal muscle relaxing agent, and bromocriptine, a dopamine agonist, can be used for a 7–10-day course with no clear evidence behind a slow taper afterwards to prevent recurrence [11]. Clinical improvement can be seen within a few days and the syndrome is usually resolved within two weeks, which was the course of our patient. Other methods of treatment such as electroconvulsive therapy have been used and may reduce mortality [12].

Prognosis of NMS has greatly improved in the past 20 years with increased recognition and diagnosis of the disorder. Mortality rates remain around 10% (5–20%) but complete recovery is seen in most patients [13]. Mortality depends on disease severity and medical complications such as renal failure. Early detection and high clinical suspicion are critical to identifying and treating both NMS and SS. It is important to identify one syndrome over the other, but this is often difficult given the many clinical overlaps. Therefore, although we are almost confident that our patient had NMS, we were cautious to still consider SS as a potential diagnosis. We removed any causative medication for either syndrome, but ultimately we treated for NMS and the patient's symptoms resolved.

## 4. Conclusion

NMS can only be diagnosed with close attention to clinical symptoms, a detailed physical exam, and a high suspicion of culprit medications. In the days of overused radiographic studies and lab tests, it is easy to overlook this clinical diagnosis. Hence, it is of utmost importance to carefully review the medication list of all admitted patients and keep a high clinical suspicion of NMS and SS in patients with a psychiatric history on medical therapy who develop high grade fevers, altered mental status, dysautonomia, elevated CPK levels in blood, and neuromuscular hyper- or hypoactivity. Furthermore, our case suggests the possible overlap between NMS and SS. Accurately differentiating between the syndromes is challenging in many clinical scenarios and therefore it may be prudent to manage both concomitantly. As more research and case studies emerge, we hope to learn more about NMS and SS and the optimal management for each.
